# Mobile Phone Addiction and Sleep Quality among Older People: The Mediating Roles of Depression and Loneliness

**DOI:** 10.3390/bs13020153

**Published:** 2023-02-10

**Authors:** Hemei Tian, Yifu Wang

**Affiliations:** Department of Psychology, Huaiyin Normal University, Huaian 223300, China

**Keywords:** mobile phone addiction, sleep quality, depression, loneliness, older people

## Abstract

Rapid social development has made the elderly increasingly dependent on mobile phones, and mobile phone addiction has a negative effect on sleep quality. The underlying mechanism between the two is unclear. This study examined the mediating role of depression and loneliness in the relationship between phone addiction and sleep quality in older adults. Mobile Phone Addiction Scale Short Version, Pittsburgh Sleep Quality Index scale, UCLA (University of California, Los Angeles)-8 Loneliness Scale, and Short Geriatric Depression Scale (GDS-15) were used to investigate 459 older adults in China. The results showed that there was a positive correlation between mobile phone addiction and sleep quality in the elderly. In addition, depression and loneliness partially mediated the relationship between mobile phone addiction and sleep quality in older adults. The current study provides new insights into the impact of mobile phone addiction on sleep quality and the importance of depression and loneliness in older adults. The limitations and significance of this study are discussed.

## 1. Introduction

With the development of science and technology and the popularity of smartphones, more and more older people use smartphones. Using mobile phones has gradually become an indispensable part of their lives. If the elderly rely heavily on mobile phones, it will have a negative impact on their emotions and behaviors [1]. Sleep is the most basic physical need of the human body. As age increases, the physical functions of older individuals become weaker and the quality of sleep decreases. The reasons for the decline of sleep quality in the elderly are complex. Exploring factors and mechanisms that affect the quality of sleep in the elderly has attracted the attention of researchers. Previous studies have confirmed in other groups that sleep quality gradually deteriorates as the degree of mobile phone addiction deepens [2,3]. However, there is little literature on the effects of mobile phone addiction on sleep quality in the elderly. Even less is known about the mechanisms underlying how mobile phone addiction affects sleep quality in older adults. In addition, previous studies have shown that depression and loneliness are common mental health problems in the elderly [4,5], which are, respectively, related to mobile phone dependence and sleep quality [6,7,8,9]. This study mainly examined the mediating effects of depression and loneliness on the relationship between mobile phone addiction and sleep quality in elderly Chinese. These findings will shed light on whether mobile phone addiction in the elderly is the cause of poor sleep and its underlying mechanism, and provide reference for future research.

### 1.1. Mobile Phone Addiction and Sleep Quality

Affected by various factors, older people have sleep difficulties to a certain extent, and many older adults suffer from sleep quality deterioration [10]. As a result of smartphones’ popularity, easy user interface, and diversified functions, the phenomenon of elderly people becoming “head-bowed parents” is also increasing. As a behavioral and psychological disorder, mobile phone addiction (MPA) can have a variety of adverse effects on the physical, psychological, and social adaptation of individuals [11]. Several studies have examined the relationship between cellphone use and sleep quality. For example, in a study of medical students, cellphone dependence was found to be associated with poor subjective sleep quality and sleep latency [12]. Another study of 1225 adults older than 18 showed that using mobile phones after lights out significantly negatively impacted adults’ sleep, specifically their sleep latency, efficiency, and quality [13]. Meanwhile, a study of 855 adults aged 18–77 reported that more time spent on social media was associated with shorter sleep duration and insomnia on weekdays [14]. Previous studies have suggested that mobile phone use affects sleep because the light emitted by the screen of electronic devices may affect the secretion of melatonin [15]. However, melatonin is a vital factor in promoting sleep [16]. Thus, there is a close relationship between excessive mobile phone use and decreased sleep quality. Previous studies have focused on adolescents, and few have revealed relationship between mobile phone addiction and sleep quality in the elderly. Therefore, we put forward the following hypothesis:

**Hypothesis** **1.** *There is a significant positive correlation between mobile phone addiction and sleep quality in the elderly.*

### 1.2. Mobile Phone Addiction, Depression, and Sleep Quality

Depression is a common mental health problem in the elderly, which has various effects on the physical and mental health of the elderly. Several studies have confirmed that depression affects the quality of sleep. Dzierzewski et al. [17] believed that depression was a predictor of sleep quality in the elderly, and subsequent studies showed a relatively stable relationship between the two. For example, Becker et al. [18] used a meta-analysis method to select related studies with elderly participants to evaluate the association between depression and sleep using a random effects method. The results found that lack of good sleep quality was significantly associated with depression in the old people. Similarly, Yu et al. [19] found that the geriatric Depression Scale score significantly correlated with sleep quality after investigating and analyzing older Asian adults.

The social displacement hypothesis suggests that communication on social networking sites replaces valuable daily social interactions with family and friends and has a negative impact on an individual’s mental health [20], potentially raising levels of depression. In other words, excessive cell phone use by the elderly may lead to poor interpersonal relationships, lack of adequate social support, experience less sense of fulfillment, and lower sense of worth, and, therefore, higher levels of depression. Chen et al. [21] believe mobile phone addiction increases distress and strengthens anxiety, depression, and other negative emotions. Li et al. [2] found a significant positive correlation between mobile phone addiction and depression through a meta-analysis of 33,650 college students. It follows that a high MPA level was a positive indicator of increased depression. Researchers have explored the relationship between mobile phone use and depression and sleep quality in college students. The results found that high frequency of mobile phone use was associated with higher levels of depression and worse sleep quality, and depression predicted worse sleep quality [22,23]. On the basis of these results, it is pointed out that depression might be a mediator between excessive smartphone use and poor sleep quality [22]. The previous literature has speculated that depression mediates the relationship between phone addiction and sleep quality, but this relationship has not been tested in older age groups. Therefore, we put forward the following hypothesis:

**Hypothesis** **2.** *Depression would mediate the relation between mobile phone addiction and the sleep quality of the elderly.*

### 1.3. Mobile Phone Addiction, Loneliness and Sleep Quality

Loneliness is a psychological feeling produced when an individual’s interpersonal relationship fails to reach the desired level, often accompanied by negative psychological experiences such as emptiness, boredom, helplessness, and depression [24,25]. Loneliness is widespread among older people as they age. An eight-year longitudinal study in the UK assessed the prevalence of autism in the elderly population and showed that about 9% of the elderly population in the UK suffered from severe loneliness, and another 30% showed some degree of loneliness [26]. Loneliness is closely linked to individual mental health. Luo et al. [27] believed that loneliness reduces sleep quality and increases morbidity and mortality. Najafi et al. [28] surveyed nursing students and found that loneliness was positively correlated with sleep quality. When exploring the relationship between loneliness and sleep quality in old people, it was found that frequent experience of loneliness increases the risk of sleep disturbance in older adults [29].

Excessive use of mobile phones can have harmful effects on individuals. That said, excessive use of smartphones and the internet may increase feelings of loneliness. According to Digital Goldilocks Hypothesis, in a society where digital media is prevalent, excessive use of network technology may replace other adaptive and meaningful social activities, thereby enhancing individual loneliness [30]. Ezoe and Toda [31] investigated medical students and found that loneliness was significantly positively correlated with mobile phone addiction. At the same time, Bhardwaj and Ashok [32] also found that mobile phone addiction was significantly related to loneliness among college students. However, further tests are needed to see whether the results can be replicated in older adults. At the same time, the above studies provide theoretical support that loneliness may mediate the relationship between cell phone addiction and sleep quality. Therefore, we put forward the following Hypothesis 3:

**Hypothesis** **3.** *Loneliness would mediate the relation between mobile phone addiction and the sleep quality of the elderly.*

### 1.4. The Present Study

This study aimed to explore the relationship between mobile phone addiction, depression, loneliness, and sleep in the elderly using a mediating analysis. Based on the previous literature, this study proposed the following hypotheses: (a) mobile phone addiction in the elderly can negatively affect sleep quality; (b) depression mediates the relationship between mobile phone addiction and sleep quality in older adults, and (c) loneliness mediates the relationship between mobile phone addiction and sleep quality in older adults. Using a dual juxtaposition mediation model, this study is the first to explore the mechanisms between mobile phone addiction and sleep in the elderly.

## 2. Method

### 2.1. Participants and Procedure

We distributed leaflets in communities of Huai‘an City and Nanjing City, Jiangsu Province, and recruited 510 older adults (43.1% male) who were over 60 years old, understood the purpose of the study, and could complete the questionnaire. Participants could receive 10 yuan after completing the questionnaire. Invalid questionnaires were excluded according to the following criteria: (a) the completion time was too short, or the survey was interrupted. Of these, 39 participants dropped out of the survey. (b) Missing data. Twelve participants did not fill in more than 50% of the items on the questionnaire and were excluded. Two of the participants had one item missing. We filled the data according to the serial average of the item [33]. Finally, a total of 51 invalid questionnaires were collected and 459 valid questionnaires were collected, of which 43.1% were male and the effective recovery rate was 90%. The study was conducted in the community by trained data collectors, and all participants gave their informed consent for inclusion prior to taking part in the study. This study was conducted in accordance with the Declaration of Helsinki, and the protocol was approved by the Ethics Committee of Huaiyin Normal University (20220311).

### 2.2. Measures

Mobile Phone Addiction Scale Short Version.

Mobile Phone Addiction Scale Short Version (SAS-SV) is a short version adapted from the Smartphone Addiction Scale (SAS) by Kwon et al. [34]. Based on the smartphone Addiction Scale (SAS), 10 out of 33 questions (e.g., “Won’t be able to stand not having a smartphone”) were selected and scored with Likert 6 points. On a scale from “strongly disagree” to “strongly agree,” higher scores indicate higher levels of phone addiction. In this study, the internal consistency test result (Cronbach’s alpha) of SAS-SV was 0.912.

Pittsburgh Sleep Quality Index (PSQI) scale.

Pittsburgh Sleep Index (PSQI), developed by Buysse et al. [35], was used to investigate the sleep status of the elderly in the past month. PSQI consists of 19 self-rated items. The items in the scoring were divided into seven components: subjective sleep quality, sleep latency, sleep time, sleep efficiency, sleep disorder, use of sleep medication, and daytime dysfunction. Each component is scored on a scale of 0–3. The higher the score, the worse the quality of sleep. In this study, the internal consistency test result (Cronbach’s alpha) of PSQI was 0.701.

UCLA (University of California, Los Angeles, CA, USA)-8 Loneliness Scale.

UCLA (University of California, Los Angeles)-8 (ULS-8) Loneliness Scale was adapted by Hays and DiMatteo [36] based on the ULS-20 Scale. There are 8 items on loneliness scale, including 6 items in positive order of “lonely” and 2 items in reverse order of “non-lonely” (item 3, “I am a person who is willing to make friends,” item 6, “I can find someone to accompany me when I am sad”). Each item is scored with a four-level frequency, and the scoring method is 1 (never), 2 (rarely), 3 (sometimes), and 4 (always). A higher total score indicates a higher level of loneliness. In this study, the internal consistency test result (Cronbach’s alpha) of PSQI was 0.751.

Short Geriatric Depression Scale (GDS-15).

In 1986, Sheikh and Yesavage [37] designed a simplified Geriatric Depression Scale (GDS-15) with 15 items based on the standard version of 30 items. The scale consists of 15 items, which are answered by “yes” or “no.” Each “yes” is worth 1 point, and “no” is worth 0 points. Items 1, 5, 7, 11, and 13 are scored in reverse (e.g., “Are you in good spirits most of the time?). A higher score indicates more depressive symptoms. In this study, the internal consistency test result (Cronbach’s alpha) of GDS-15 was 0.784.

### 2.3. Statistical Analyses

SPSS 26.0 and Process v3.4 were used for statistical analysis. The SPSS software was used to perform descriptive statistics and correlation analysis for all variables, and Process software was used to explore potential relationships between the variables studied. In process v3.4, the Bootstrap sampling method was used to test the mediating effect [38]. Confidence intervals without zero indicated significant corresponding effects, *p* < 0.05 statistically significant.

## 3. Results

### 3.1. Preliminary Analyses

Pearson correlation analysis showed that cell phone addiction was positively associated with depression, loneliness, and sleep quality in older adults. In other words, the higher the level of cell phone addiction, the worse the quality of sleep for older adults. Summary descriptive statistics of the main variables and their correlations are shown in Table 1.

### 3.2. Mediational Analysis

**Figure 1:**
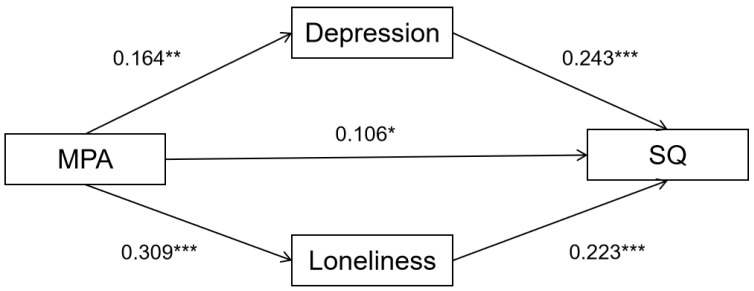
Model with Depression and Loneliness as mediators in the relation of mobile phone addiction (MPA) with sleep quality (AQ). * *p* < 0.05; ** *p* < 0.01; *** *p* < 0.001.

The mediating effect program compiled by Hayes [38] was used to calculate the mediating effect by the Bootstrap method with deviation correction, Bootstrap samples was 5000, and the confidence interval was 95%. The mediating effect analysis was performed with cell phone addiction as an independent variable, loneliness and depression as mediating variables, sleep quality as a dependent variable, and age and gender as control variables. The results for the path coefficients are shown in Figure 1 and Table 2.

The results of mediating effect test showed that, without adding mediating variables, mobile phone addiction significantly positively affected sleep quality (*β* = 0.22, *p* < 0.001). After adding mediating variables, mobile phone addiction still had a significant positive effect on sleep quality (*β* = 0.106, *p* < 0.05), loneliness significantly positively affected sleep quality (*β* = 0.223, *p* < 0.001), depression significantly positively affected sleep quality (*β* = 0.243, *p* < 0.001), and mobile phone addiction had a significant positive effect on depression (*β* = 0.164, *p* < 0.001), mobile phone addiction significantly positively affected loneliness (*β* = 0.309, *p* < 0.001).

In addition, the effect value of the direct path of mobile phone addiction on sleep quality was 0.106, and the effect value of the indirect path of depression and loneliness was 0.04 and 0.069, respectively. The upper and lower limits of the Bootstrap 95% confidence intervals for the above paths do not contain 0, indicating that the above paths were significant. The results show that mobile phone addiction directly and positively affects sleep quality, and indirectly through depression and loneliness. Direct effects accounted for 49.302 percent of the total, and indirect effects of depression and loneliness accounted for 18.605 percent and 32.093 percent of the total. The effects of mobile phone addiction on sleep quality are shown in Table 3.

## 4. Discussion

In order to improve sleep quality, it is of great significance to understand the influencing factors and internal mechanisms of sleep quality. Therefore, this study examined the relationship between mobile phone addiction and sleep quality in the elderly and constructed a parallel mediation model to investigate the indirect effects of depression and loneliness on the relationship between mobile phone addiction and sleep quality in the elderly. The findings suggested that cell phone addiction has a direct effect on sleep quality in older adults, as well as an indirect effect through depression and anxiety.

### 4.1. Mobile Phone Addiction and Sleep Quality in Older Adults

The result showed a significant positive correlation between mobile phone addiction and sleep quality in the elderly, validating the first hypothesis. That is, the degree of mobile phone addiction is significantly positively correlated with the sleep quality of the elderly, which is consistent with the results of previous studies in other groups Liu et al., 2017 [39]. Based on the microanalytic model of insomnia proposed by Morin [40]. This model focuses on the interaction between the organism, the environment, and various variables in a particular scenario. According to Morin, excessive wakefulness is a significant cause of poor sleep quality. Whether it is behavioral, physical, or verbal hyperarousal, it is not conducive to the normal progression of sleep. Excessive cell phone use includes a variety of cognitive, behavioral, and physiological stimuli that increase arousal levels and lead to decreased sleep quality. For example, physical hyperarousal may occur when reading information on a mobile phone, or verbal hyperarousal may occur when chatting with friends online. In this way, over-reliance on mobile phones has a negative impact on the quality of sleep in older people.

### 4.2. Mediating Effects of Depression

The results of this study suggest that depression mediates the relationship between cell phone addiction and sleep quality in older adults, validating Hypothesis 2. First of all, the higher the degree of mobile phone addiction in the elderly, the higher the level of depression, which is consistent with previous research results [6]. Although some previous studies have suggested that the appropriate use of mobile phones in the elderly can reduce the risk of depression [41], it is obvious that if the elderly are excessively addicted to mobile phones, they are prone to lose control, fall into the “cognitive cycle” unconsciously, and produce “subconscious thinking.” This mode of thinking, once formed, is prone to depression. As a result, uncontrolled cell phone use may contribute to depression in the elderly. Secondly, there was a significant positive correlation between depression and sleep quality. That is, higher levels of depression are associated with lower sleep quality. This study builds on the previous literature by replicating this finding in a sample of older Chinese adults. The results are in line with the findings of previous studies, such as Maglione et al. [42], which also found a strong association between more severe depressive symptoms and poor sleep quality in older adults. Older adults who are addicted to their phones are more likely to develop depressive symptoms such as decreased energy, inattention, and daytime drowsiness, which can further disrupt sleep rhythms and reduce sleep quality. The mediating effect results showed that the mediating effect size of depression between cell phone addiction and sleep quality was 18.605 percent of the total effect size, indicating that depression is a mediating variable in the effects of cell phone addiction on sleep quality in older adults.

### 4.3. The Mediating Role of Loneliness

The results of this study suggest that loneliness mediates the relationship between cell phone addiction and sleep quality in the elderly, validating hypothesis 3. First, the higher the degree of mobile phone addiction in the elderly, the higher the level of loneliness, which is consistent with the research results in other groups [43]. According to general cognition, the elderly can increase communication with the outside world by using mobile phones, which is conducive to eliminating loneliness [44]. However, the results of this study suggest that there is a significant positive correlation between cell phone addiction scores and loneliness among older adults. It can be seen that excessive use of mobile phones is not conducive to increasing communication with the outside world. The more the elderly escape from reality and indulge in behavior on mobile social platforms, the more likely they are to feel lonely. Because excessive reliance on virtual platforms will inevitably reduce interpersonal communication behavior in real life when the psychological support, in reality, is missing or insufficient, they try to replace the real support and emotional comfort through the virtual support, which is often ineffective [45]. Second, there was a significant positive correlation between loneliness and sleep quality, that is, higher levels of loneliness were associated with poorer sleep quality. This is consistent with the results of previous studies [29]. As they grow older, older adults lose their ability to maintain attention on their external environment and become sensitive to stimuli. Once they experience a sense of loss, helplessness, and loneliness as the number of people around them they can talk to decreases, their sleep quality suffers. The mediating effect showed that the mediating effect size of loneliness between mobile phone addiction and sleep quality accounted for 32.093 of the total effect size, which was slightly higher than mediating effect of depression. These results suggest that loneliness is also an important mediating variable in the influence of mobile phone addiction on sleep quality in the elderly. Overall, depression and loneliness partially mediate the relationship between mobile phone addiction and sleep quality in older adults.

## 5. Limitations and Implications

The present study has several limitations. The first is due to the cross-sectional nature of our study design. Therefore, conclusions about causality should be interpreted with caution. Future studies could use longitudinal designs to extend the current findings. Second, the study was conducted in the context of Chinese culture, and the sample in the study was limited to a single province. Future research should expand the sampling range to better validate the findings. Third, the relationship between mobile phone addiction and depression is complex. Because it has been suggested that a person with depressive symptoms may turn to social networking sites to gain social approval by gaining likes and followers, depression may be an antecedent of mobile phone addiction [46]. In other words, there may be a reverse causation between mobile phone addiction and depression in the elderly. Similarly, there may be a reverse causation between cell phone addiction, loneliness, and sleep quality. This study had a cross-sectional design and was not able to investigate reverse causality between variables. In the future, longitudinal and experimental studies can be carried out to explore this in greater depth. In addition, the current study showed that depression and loneliness partially mediated the association between mobile phone addiction and sleep quality in older adults, suggesting that other variables may also mediate the association between mobile phone addiction and sleep quality in older adults. Similarly, some demographic variables, such as marital status, are likely to have a moderating effect. Therefore, the complex relationship between cell phone addiction and sleep quality in the elderly needs further investigation.

Most previous research on cell phone addiction has focused on the adult population, and there have been few studies of cell phone addiction in the elderly. In some studies of cell phone use among older adults, the results have shown that cell phone use is beneficial for older adults. But the results of this study provide some empirical support for the displacement hypothesis [47], which holds that the damage of technology is proportional to exposure. That is, the higher the level of cell phone addiction, the greater the negative emotions and behaviors of the elderly. As the Chinese idiom goes, “too much of a good thing is not enough”, which means it is inappropriate to overdo something. The present work is the first attempt to examine the mediating role of depression and loneliness in the relationship between mobile phone addiction and sleep quality in the elderly, providing empirical support for the study of the relationship between mobile phone addiction and sleep quality in the elderly. The correlation between mobile phone addiction and sleep quality in the elderly and the underlying mechanisms have been explained to some extent.

In addition, the present study has important practical implications. First, children or other partners must help older adults understand the adverse effects of cell phone addiction on sleep quality and encourage them to engage in activities that are good for their physical and mental health to reduce their reliance on their phones. Second, the study found that depression is a mediator of phone addiction affecting sleep quality, so reducing depression in the elderly is significant. Previous studies have found that using positive reappraisal cognitive strategies in the elderly can increase the ability to regulate emotions so that their depression symptom scores are lower [48]. In addition, studies have also found that more people participate in sports and other meaningful social activities [49,50], which can reduce depressive symptoms in older adults by providing them with social support and reducing the production of the stress hormone cortisol. Third, loneliness also mediates between phone addiction and sleep quality. So, older people need to try some ways to reduce their loneliness. Elias et al. [51] found that group recall therapy can effectively relieve loneliness because individuals can obtain information from memories to combat loneliness. Some researchers believe that the loneliness experience of the elderly can be reduced through community publicity or the organization of community activities [52].

Mobile phones do bring many conveniences to our lives, but the results of this study suggest that, in an older age group, over-reliance on cell phones increases the risk of depression and loneliness and has a negative impact on sleep quality. In other words, mobile phone addiction in older people has a certain amount of negative physical and psychological effects. This result suggests that we should be wary of the behaviour of older people who become addicted to mobile phones during their lives. For children, more time should be devoted to quality elderly company. In communities, activity centers for the elderly can be set up to hold various activities to enrich the leisure life of the elderly. In short, reducing the dependence of older people on mobile phones and improving the quality of their spiritual lives requires the joint involvement of families and society.

## 6. Conclusions

In conclusion, this study is the first to analyze the mediating effects of depression and loneliness on the association between mobile phone addiction and sleep quality in older adults. These findings contribute to a better understanding of the causes and underlying mechanisms underlying the decline in sleep quality in older adults. According to the results of this study, mobile phone addiction directly impacts sleep quality in the elderly and indirectly impacts sleep quality through two mediating pathways: depression and loneliness. The results of this study suggest that smartphone use is a double-edged sword, and that over-reliance on cell phones has become a cause of emotional and behavioral problems in the elderly. Importantly, the results of this study serve as a reminder to pay attention to the frequency of cell phone use in older adults, and to pay attention to enriching the leisure life of older adults so as to improve their emotional and sleep problems.

## Figures and Tables

**Table 1:** Descriptive statistics and correlation coefficients for the variables.

	M	SD	Age	Gender	MPA	Depression	Loneliness	SQ
Age	63.44	3.958	-	−0.027	−0.092 *	−0.024	0.052	−0.002
Gender				-	−0.030	0.031	−0.083	−0.007
MPA	29.595	9.786			-	0.164 **	0.309 **	0.215 **
Depression	19.024	3.196				-	0.468 **	0.365 **
Loneliness	16.050	3.918					-	0.370 **
SQ	7.913	3.674						-

*N* = 459. * *p* < 0.05; ** *p* < 0.01. MPA, mobile phone addiction, SQ, sleep quality.

**Table 2:** The regression analysis of variable relationship.

Outcome Variables	Independent Variables	*R*	*R2*	*β*	t
SQ	MPA	0.214	0.046	0.215	4.488 ***
Depression	MPA	0.164	0.027	0.164	3.348 **
Loneliness	MPA	0.309	0.096	0.309	6.693 ***
SQ	Depression	0.440	0.194	0.243	4.403 ***
	Loneliness			0.223	4.597 ***
	MPA			0.106	2.271 *

* *p* < 0.05; ** *p* < 0.01; *** *p* < 0.001.

**Table 3:** Effects of mobile phone addiction on sleep quality.

Model Path	Effect Size	Boot SE	BootLLCI	BootULCI	Proportion of Total Effect (%)
Total effect	0.215	0.048	0.121	0.309	
Direct effect	0.106	0.047	0.014	0.197	49.302%
Total indirect effect	0.109	0.023	0.068	0.159	50.698%
MPA-Depression-SQ	0.040	0.015	0.016	0.160	18.605%
MPA-Loneliness-SQ	0.069	0.018	0.039	0.109	32.093%

*N* = 459. Bootstrap sample size = 5000. *CI*, confidence interval; *LL*, lower limit; *UL*, upper limit.

## Data Availability

The data presented in this study are available on request from the corresponding author.

## References

[1] Subramanyam A.A., Singh S., Raut N.B. (2018). Mobile phone use in the elderly: Boon or bane?. J. Geriatr. Ment. Health.

[2] Li Y., Li G., Liu L., Wu H. (2020). Correlations between mobile phone addiction and anxiety, depression, impulsivity, and poor sleep quality among college students: A systematic review and meta-analysis. J. Behav. Addict..

[3] Sahin S., Ozdemir K., Unsal A., Temiz N. (2013). Evaluation of mobile phone addiction level and sleep quality in university students. Pak. J. Med. Sci..

[4] Dahlberg L., McKee K.J., Frank A., Naseer M. (2022). A systematic review of longitudinal risk factors for loneliness in older adults. Aging Ment. Health.

[5] Paudel M., Taylor B.C., Ancoli-Israel S., Blackwell T., Maglione J.E., Stone K., Redline S., Ensrud K.E. (2013). Sleep disturbances and risk of depression in older men. Sleep.

[6] Alhassan A.A., Alqadhib E.M., Taha N.W., Alahmari R.A., Salam M., Almutairi A.F. (2018). The relationship between addiction to smartphone usage and depression among adults: A cross sectional study. BMC Psychiatry.

[7] McHugh J.E., Lawlor B.A. (2013). Perceived stress mediates the relationship between emotional loneliness and sleep quality over time in older adults. Br. J. Health Psychol..

[8] Liu X., Wang C., Qiao X., Si H., Jin Y. (2021). Sleep quality, depression and frailty among Chinese community-dwelling older adults. Geriatr. Nurs..

[9] Wilson C. (2018). Is it love or loneliness? Exploring the impact of everyday digital technology use on the wellbeing of older adults. Ageing Soc..

[10] Foley D.J., Monjan A.A., Brown S.L., Simonsick E.M., Wallace R.B., Blazer D.G. (1995). Sleep complaints among elderly persons: An epidemiologic study of three communities. Sleep.

[11] Goswami V., Singh D.R. (2016). Impact of mobile phone addiction on adolescent’s life: A literature review. Int. J. Home Sci..

[12] Ibrahim N.K., Baharoon B.S., Banjar W.F., Jar A.A., Ashor R.M., Aman A.A., Al-Ahmadi J.R. (2018). Mobile phone addiction and its relationship to sleep quality and academic achievement of medical students at king abdulaziz university, Jeddah, Saudi Arabia. J. Res. Health Sci..

[13] Lastella M., Rigney G., Browne M., Sargent C. (2020). Electronic device use in bed reduces sleep duration and quality in adults. Sleep Biol. Rhythm..

[14] Bhat S., Pinto-Zipp G., Upadhyay H., Polos P.G. (2018). “To sleep, perchance to tweet”: In-bed electronic social media use and its associations with insomnia, daytime sleepiness, mood, and sleep duration in adults. Sleep Health.

[15] Exelmans L., Van den Bulck J. (2016). Bedtime mobile phone use and sleep in adults. Soc. Sci. Med..

[16] Claustrat B., Leston J. (2015). Melatonin: Physiological effects in humans. Neurochirurgie.

[17] Dzierzewski J.M., Mitchell M., Rodriguez J.C., Fung C.H., Jouldjian S., Alessi C.A., Martin J.L. (2015). Patterns and predictors of sleep quality before, during, and after hospitalization in older adults. J. Clin. Sleep Med..

[18] Becker N.B., Jesus S.N., Joao K.A., Viseu J.N., Martins R.I. (2017). Depression and sleep quality in older adults: A meta-analysis. Psychol. Health Med..

[19] Yu J., Rawtaer I., Fam J., Jiang M.J., Feng L., Kua E.H., Mahendran R. (2016). Sleep correlates of depression and anxiety in an elderly A sian population. Psychogeriatrics.

[20] Bessiere K., Kiesler S., Kraut R., Boneva B.S. (2008). Effects of Internet use and social resources on changes in depression. Inf. Community Soc..

[21] Chen L., Yan Z., Tang W., Yang F., Xie X., He J. (2016). Mobile phone addiction levels and negative emotions among Chinese young adults: The mediating role of interpersonal problems. Comput. Hum. Behav..

[22] Demirci K., Akgönül M., Akpinar A. (2015). Relationship of smartphone use severity with sleep quality, depression, and anxiety in university students. J. Behav. Addict..

[23] Kaya F., Bostanci Daştan N., Durar E. (2021). Smart phone usage, sleep quality and depression in university students. Int. J. Soc. Psychiatry.

[24] Kim J.H. (2018). Psychological issues and problematic use of smartphone: ADHD’s moderating role in the associations among loneliness, need for social assurance, need for immediate connection, and problematic use of smartphone. Comput. Hum. Behav..

[25] Rokach A. (1988). The experience of loneliness: A tri-level model. J. Psychol..

[26] Victor C.R., Bowling A. (2012). A longitudinal analysis of loneliness among older people in Great Britain. J. Psychol..

[27] Luo Y., Hawkley L.C., Waite L.J., Cacioppo J.T. (2012). Loneliness, health, and mortality in old age: A national longitudinal study. Soc. Sci. Med..

[28] Najafi F., Saravi F.K., Navidian A., Raeisi S.M. (2018). The relationship between internet addiction, loneliness and sleep quality among students of nursing and midwifery faculty. Zahedan J. Res. Med. Sci..

[29] Griffin S.C., Williams A.B., Mladen S.N., Perrin P.B., Dzierzewski J.M., Rybarczyk B.D. (2020). Reciprocal effects between loneliness and sleep disturbance in older Americans. J. Aging Health.

[30] Wang K., Frison E., Eggermont S., Vandenbosch L. (2018). Active public Facebook use and adolescents’ feelings of loneliness: Evidence for a curvilinear relationship. J. Adolesc..

[31] Ezoe S., Toda M. (2013). Relationships of loneliness and mobile phone dependence with internet addiction in japanese medical students. Open J. Prev. Med..

[32] Bhardwaj M., Ashok MS J. (2015). Mobile phone addiction and loneliness among teenagers. Int. J. Indian Psychol..

[33] Newman D.A. (2014). Missing data: Five practical guidelines. Organ. Res. Methods.

[34] Kwon M., Kim D.-J., Cho H., Yang S. (2013). The Smartphone Addiction Scale: Development and Validation of a Short Version for Adolescents. PLoS ONE.

[35] Buysse D.J., Reynolds C.F., Monk T.H., Berman S.R., Kupfer D.J. (1989). The Pittsburgh Sleep Quality Index: A new instrument for psychiatric practice and research. Psychiatry Res..

[36] Hays R.D., DiMatteo M.R. (1987). A short-form measure of loneliness. J. Personal. Assess..

[37] Sheikh J.I., Yesavage J.A. (1986). Geriatric Depression Scale (GDS): Recent evidence and development of a shorter version. Clin. Gerontol. J. Aging Ment. Health.

[38] Hayes A.F. (2018). Partial, conditional, and moderated moderated mediation: Quantification, inference, and interpretation. Commun. Monogr..

[39] Liu Y., Li T., Guo L., Zhang R., Feng X., Liu K. (2017). The mediating role of sleep quality on the relationship between perceived stress and depression among the elderly in urban communities: A cross-sectional study. Public Health.

[40] Morin C.M. (1993). Insomnia: Psychological assessment and management.

[41] Yoshany N., Mazloomy-Mahmoodabad S.S., Mihanpour H., Seyed-khameshi S.S., Gerayllo S., Nabil A. (2020). The Role of Using Smartphones on Depression Rate in Retired Elderly. Iran. J. Health Educ. Health Promot..

[42] Maglione J.E., Ancoli-Israel S., Peters K.W., Paudel M.L., Yaffe K., Ensrud K.E., Stone K.L. (2012). Depressive symptoms and subjective and objective sleep in community-dwelling older women. J. Am. Geriatr. Soc..

[43] Xu J. (2017). Research on the relationship among phone addiction, social anxiety and loneliness in high school students. Open J. Soc. Sci..

[44] Jarvis M.A., Chipps J., Padmanabhanunni A. (2019). “This phone saved my life”: Older persons’ experiences and appraisals of an mHealth intervention aimed at addressing loneliness. J. Psychol. Afr..

[45] Shen X., Wang J.L. (2019). Loneliness and excessive smartphone use among Chinese college students: Moderated mediation effect of perceived stressed and motivation. Comput. Hum. Behav..

[46] Hartanto A., Quek F.Y., Tng G.Y., Yong J.C. (2021). Does social media use increase depressive symptoms? A reverse causation perspective. Front. Psychiatry.

[47] Neuman S.B. (1988). The displacement effect: Assessing the relation between television viewing and reading performance. Read. Res. Q..

[48] Garnefski N., Kraaij V. (2006). Relationships between cognitive emotion regulation strategies and depressive symptoms: A comparative study of five specific samples. Personal. Individ. Differ..

[49] Fiske A., Wetherell J.L., Gatz M. (2009). Depression in older adults. Annu. Rev. Clin. Psychol..

[50] Wrosch C., Schulz R., Miller G.E., Lupien S., Dunne E. (2007). Physical health problems, depressive mood, and cortisol secretion in old age: Buffer effects of health engagement control strategies. Health Psychol..

[51] Elias SM S., Neville C., Scott T. (2015). The effectiveness of group reminiscence therapy for loneliness, anxiety and depression in older adults in long-term care: A systematic review. Geriatr. Nurs..

[52] Grenade L., Boldy D. (2008). Social isolation and loneliness among older people: Issues and future challenges in community and residential settings. Aust. Health Rev..

